# Niche-based assembly of bacterial consortia on the diatom *Thalassiosira rotula* is stable and reproducible

**DOI:** 10.1038/s41396-020-0631-5

**Published:** 2020-03-23

**Authors:** Julian Mönnich, Jan Tebben, Jennifer Bergemann, Rebecca Case, Sylke Wohlrab, Tilmann Harder

**Affiliations:** 1 Marine Chemistry, Department of Chemistry and Biology, University of Bremen, 28359 Bremen, Germany; 2 Section Ecological Chemistry, Alfred Wegener Institute, Helmholtz Centre for Polar and Marine Research, 27570 Bremerhaven, Germany; 3 Department of Biological Sciences, University of Alberta, Edmonton, AB Canada; 4 Singapore Centre for Environmental Life Sciences Engineering (SCELSE), Nanyang Technological University, Singapore, 637551 Singapore; 5 Helmholtz Institute for Functional Marine Biodiversity, 23129 Oldenburg, Germany

**Keywords:** Environmental microbiology, Microbial ecology

## Abstract

With each cell division, phytoplankton create new space for primary colonization by marine bacteria. Although this surface microenvironment is available to all planktonic bacterial colonizers, we show the assembly of bacterial consortia on a cosmopolitan marine diatom to be highly specific and reproducible. While phytoplankton–bacteria interactions play fundamental roles in marine ecosystems, namely primary production and the carbon cycle, the ecological paradigm behind epiphytic microbiome assembly remains poorly understood. In a replicated and repeated primary colonization experiment, we exposed the axenic diatom *Thalassiosira rotula* to several complex and compositionally different bacterial inocula derived from phytoplankton species of varying degrees of relatedness to the axenic *Thalassiosira* host or natural seawater. This revealed a convergent assembly of diverse and compositionally different bacterial inocula, containing up to 2071 operational taxonomic units (OTUs), towards a stable and reproducible core community. Four of these OTUs already accounted for a cumulative abundance of 60%. This core community was dominated by Rhodobacteraceae (30.5%), Alteromonadaceae (27.7%), and Oceanospirillales (18.5%) which was qualitatively and quantitatively most similar to its conspecific original. These findings reject a lottery assembly model of bacterial colonization and suggest selective microhabitat filtering. This is likely due to diatom host traits such as surface properties and different levels of specialization resulting in reciprocal stable-state associations.

## Introduction

Marine phytoplankton is pivotal in fixing and converting atmospheric CO_2_ into biological matter. The organic matter enters the marine food web and is partially exported to the ocean floor, supporting global marine biological and geochemical processes as a consequence [1]. These processes are largely fueled by close interactions between phytoplankton and bacteria and driven by specific bacterial enzymatic capabilities as well as reciprocal needs of bacteria and phytoplankton for essential trace elements, micro-, and macronutrients [2, 3]. Experimental evidence for such interactions is demonstrated for the utilization and acquisition of bacterially produced B-vitamins [4] and essential trace metal ions by diatoms [5]. The presumed vitamin B_12_ auxotrophy of many phytoplankton is confirmed in more than 50% of investigated stramenopiles [6].

In return for vitamins and other cofactors, phytoplankton offer an organic carbon source to heterotrophic bacteria through cell wall-associated macromolecules and sequestration of photosynthetic products [7]. Diatoms, for example, release up to 5% of their primary production as photosynthate [8]. A high proportion of this dissolved organic carbon consists of high-molecular weight (HMW) components such as polysaccharides [9]. Recent research using phototroph–heterotroph co-culture experiments under nutrient-amended and natural seawater conditions suggests that phytoplankton and bacterial heterotrophs do not compete for the same limited resources, but rather benefit from each other because of very different levels of specialization resulting in complementary associations in long-term stable-state systems [10]. One of these studies revealed feedback loops of bacteria producing algal growth hormones from diatom-derived precursor molecules in exchange for diatom-excreted organosulfur molecules, which in turn stimulate bacterial excretion of ammonia subsequently fueling diatom growth [11]. Despite these sophisticated examples of reciprocal metabolic exchange, the role of these compounds in organismal interactions and in the assembly of bacterial consortia on phytoplankton are poorly studied [7].

The exudation of organic photosynthate molecules by phytoplankton involves passive diffusion driven by a steep concentration gradient between the phytoplankton cell and its surroundings. The diffusive boundary layer causing this gradient is the algal phycosphere [12], which defines the location for direct interactions between microalgae and bacteria. It is largely made up of HMW polysaccharides. Depending on their monomer composition, type and degree of branching and ultrastructure, phytoplankton exopolysaccharides represent highly diverse and often plankton species-specific chemical compounds [13]. These molecules are crucial attractants for heterotrophic bacteria [14]. For bacteria to utilize these exopolysaccharides requires specific carbohydrate-active enzymes [15] encoding the machinery for polysaccharide detection, hydrolysis, and uptake. Such enzymes are localized within gene clusters referred to as polysaccharide utilization loci (PULs). As no single bacterium is adequately specialized to utilize the suite of complex phytoplankton exopolysaccharides, commensal and mutualistic associations of different heterotrophic bacteria with complementary PULs are assumed to co-occur in the phycosphere. This in turn gives rise to the emerging concept of plankton-specific associated consortia [14, 16].

The above examples indicate that an epiphytic bacterial community is important to normal phytoplankton function and, by extension, the ecology of the habitat in which they exist. Indeed, several recent microbiome studies of diverse phytoplankton suggest that marine and fresh water phytoplankton harbor unique bacterial consortia which are consistent within phytoplankton species and across temporal scales [16–19]. Yet, some phytoplankton do not harbor a core set of associated bacteria [20]. In these cases, the sampling time and location are suggested to be more decisive factors determining the associated microbiome than phytoplankton species affiliation [21].

The principles underlying the assembly of complex epiphytic microbial communities have been an issue of long-standing concern to the field of marine microbial ecology. Contrary to microbiome studies of marine microalgae, there are detailed and spatiotemporally replicated studies of epiphytic communities on macroalgal surfaces. The green seaweed *Ulva australis*, for example, harbors a highly specific associated bacterial community distinct from that of surrounding seawater but with a high degree of variability among *U. australis* individuals [22]. These observations reject the hypothesis of a stable, algal species-specific core microbiome and suggest that a large number of bacterial species colonize this macroalgal surface. Burke et al. [22] linked this conclusion to the redundancy hypothesis [23], which assumes that more than one species is capable of performing a specific role within an ecosystem. Yet, on its own, functional redundancy did not account for observations of both selectiveness and variability, suggesting additional selective mechanisms determining the epiphytic bacterial assemblage on the seaweed. The authors reconciled their observations with the lottery hypothesis [24, 25], an ecological assembly model originally developed to explain the coexistence of reef fish species, that departs from the traditional niche-based view. This theory asserts that species with similar trophic abilities will occupy space within an ecosystem based on stochastic recruitment, i.e., whoever gets there first wins the lottery for space. In *U. australis*, this model entails that a guild of certain bacteria, all of which possess the necessary genetic abilities to colonize and metabolic requirements to live on the thallus surface, is functionally redundant [26]. These considerations are particularly pertinent in the context of microbial community ecology, given the frequent genetic exchange among taxonomically distinct bacteria through horizontal gene transfer, resulting in a high degree of genomic and functional coherence.

There is currently no unifying scheme or theory for bacterial community assembly on marine phytoplankton that satisfies the diverse observations spanning the spectrum from distinct core communities on phylogenetically distant microalgae to spatially and/or temporally diverse communities on conspecific microalgae. Notably, studies addressing bacterial community assembly resulting from interactions between two microscopic players (i.e., phytoplankton and seawater bacteria) are challenging due to the microscopic scale at which they occur. The only studies to date, comparing bacterial assemblages on individual cells of congeneric diatoms obtained from natural waters, found that recovered bacterial phylotypes were extremely diverse and rarely shared across individual diatom cells [27, 28], potentially supporting a lottery assembly model.

We conducted a series of independent and replicated co-culture experiments to unravel patterns of bacterial community assembly on a globally important, bloom-forming marine diatom genus, *Thalassiosira* [29, 30], specifically a North Sea isolate of *T. rotula*. The diatom was rendered axenic and cultured under micronutrient-poor conditions in the absence of B-vitamins. These axenic *T. rotula* cultures were inoculated with complex bacterial communities detached from conspecific (from the same species), congeneric (from the same genus), and heterospecific (from a different species) co-occurring diatom species and seawater bacterioplankton. Once these co-cultures reached mid-exponential growth phase, the newly established, primary *T. rotula* microbiomes were sampled and sequenced for biostatistical analyses.

## Materials and methods

### Phytoplankton collection, species characterization, and culture maintenance

Monoclonal phytoplankton batch cultures were established by isolating single cells or chains of microalgae from seawater collected at the island of Helgoland (54°11′03′′ N, 7°54′00′′ E). The isolates were grown in 12-well plates with filter-sterilized artificial seawater medium (ESAW) containing essential trace metals and vitamins [31] with the exception of *T. rotula*_A17, which was isolated and maintained in vitamin-depleted ESAW. Pure and established cultures were transferred to 25 mL culture flasks and maintained at 15 °C and a 12 h light/12 h dark diurnal cycle (30–70 µmol m^−2^ s^−1^). Every week, aliquots of batch cultures were transferred into new medium at a concentration of 2000 cells mL^−1^ to maintain healthy and exponentially growing cultures. Taxonomic identities of *T. rotula* strain S16 (isolated in spring 2016, in the following labeled *T. rotula*_S16), *T. rotula* strain A17 (isolated in autumn 2017, in the following labeled *T. rotula*_A17), and axenic *T. rotula*_S16 (see below), were assigned by sequence similarity analysis of a fragment of the small and large subunit of the 18S and 28S ribosomal RNA gene (see Supplementary section). Briefly, algal pellets were processed with the DNeasy Powersoil kit (Qiagen, Germany) according to the manufacturer’s instructions. PCR was performed with specific primers [32, 33]. The other diatom isolates were identified based on morphological characteristics [34] resulting in the assignment of *Ditylum brightwellii* and *Cylindrotheca closterium* (isolated in spring 2016). Two other *Thalassiosira* species, *T. weissflogii* (CCMP 3365) and *T. pseudonana* (CCMP 996) were obtained from the National Centre for Marine Algae and Microbiota (NCMA at Bigelow Laboratory, USA) and maintained under the same conditions described above.

In growth experiments, diatom growth was monitored daily in black 96-well polystyrene microplates with clear bottom (Greiner Bio-One, Germany) by measurements of relative fluorescence units (RFU) of chlorophyll using a plate reader (FLUOstar Omega, BMG, Germany) at optical filters settings of *λ*_ex_ 440–80 nm and *λ*_em_ 640–80 nm. In Experiment I diatom growth and performance were further monitored by pulsed-amplitude-modulation fluorometry (Water-PAM, Walz, Germany) by measurement of the minimal (*F*_0_) and maximal (*F*_m_) dark fluorescence [35]. Briefly, samples were taken 5–7 h into the light cycle and diluted in ESAW to be within the PAM detection range. The photosystem II (PSII) potential quantum yield (*F*_v_/*F*_m_) is the normalized ratio of *F*_0_ and *F*_m_ ((*F*_m_ − *F*_0_)/*F*_m_ = *F*_v_/*F*_m_) and represents the maximum potential quantum efficiency [36]. The chlorophyll fluorescence readings were correlated with exact numbers of Lugol-fixed diatom cells enumerated under the microscope.

### Establishment of an axenic *T. rotula* culture

The *T. rotula*_S16 culture was rendered axenic according to [37] with modifications. Briefly, 40 mL diatom culture was harvested at mid-exponential growth and gravity filtered onto 3-µm pore-size polycarbonate Nucleopore track-etched membrane filters (Whatman, Germany) in a sterile glass vacuum filter device. Diatoms on the filter membrane were treated sequentially with (i) 150 mL sterile ESAW, (ii) 50 mL sterile ESAW containing 20 µg mL^−1^ Triton-X 100 (Sigma Aldrich, Germany), and (iii) 150 mL sterile ESAW. The treated cells were transferred into 100 mL sterile ESAW containing the antibiotics streptomycin (50 µg mL^−1^), gentamicin (67 µg mL^−1^), ciprofloxacin (20 µg mL^−1^), chloramphenicol (2.2 µg mL^−1^), and ampicillin (100 µg mL^−1^) (Sigma, Germany) and incubated for 2 days under the temperature and light settings above. Subsequently, the entire procedure was repeated twice to render the culture axenic. The axenic culture was again gravity filtered, filter washed and transferred into sterile ESAW without antibiotics at ca. 2000 cells mL^−1^ and grown under the temperature and light settings above. The culture was regularly checked for axenicity by nucleic acid staining with 4′,6-diamidino-2-phenylindole (DAPI, Thermo Fisher Scientific, Germany) under the fluorescence microscope. In parallel, the diatom culture was regularly inoculated into marine broth to check for bacterial growth and contamination.

### Preparation of *T. rotula* for co-culture with a bacterial inoculum

Prior to inoculating diatoms with a defined bacterial consortium, the axenic *T. rotula*_S16 culture was depleted of vitamins. This was done by gravity filtration of 40 mL axenic culture at mid-exponential phase, rinsing the filter three times with 100 mL ESAW without vitamin supplements (ESAW^-vit^). An aliquot of the axenic, vitamin-replete *T. rotula*_S16 culture was transferred to new ESAW^-vit^ at 2000 cells mL^−1^ and maintained under temperature and light settings as described above until mid-exponential phase. These transfers were repeated until the culture no longer grew in comparison with an axenic and vitamin-replete *T. rotula*_S16 culture. The axenic and vitamin-deplete *T. rotula*_S16 culture was immediately used as a recipient (in the following termed acceptor culture) of defined bacterial inocula.

### Preparation of the bacterial inocula

The non-axenic diatom cultures were transferred from ESAW to ESAW^-vit^ for 2 months prior to their use as a source of the bacterial inoculum. Briefly, 40 mL culture aliquots (*n* = 3) were gravity filtered through 3-µm (0.6 µm for *T. pseudonana)* pore-size polycarbonate membranes, rinsed with 100 mL ESAW^-vit^ and incubated in 40 mL ESAW^-vit^ for 2 days, after which the procedure was repeated twice. The logic behind this procedure was to flush free-living, opportunistic bacteria out of the cultures. This strategy relied on the rationale that diatom-associated bacteria coexist with dissociated conspecifics. The final 40 mL filtrates of each diatom culture were quantified by fluorescence microscopy of DAPI-stained bacterial cells and immediately used as inoculum. The filter membranes were transferred into SL1 lysis buffer (NucleoSpin Soil® kit, Macherey Nagel, Germany) and stored at −20 °C for subsequent sequencing analyses of each diatom-associated bacterial community. Replicates of the *T. rotula*_S16 acceptor cultures were processed accordingly.

### Co-culture experiment

Aliquots of the axenic, vitamin-deplete *T. rotula*_S16 acceptor culture were adjusted to 2000 cells mL^−1^ in ESAW^-vit^ and spiked with bacterial inocula at a ratio of 1:100 diatom:bacterial cells obtained from (i) conspecific non-axenic *T. rotula*_S16 and *T. rotula*_A17, (ii) congeneric *T. weissflogii* and *T. pseudonana*, (iii) heterospecific *D. brightwellii* and *C. closterium*, and (v) fresh seawater from Helgoland obtained in autumn 2017 (54°11.3′ N, 7°54.0′ E). An axenic, vitamin-deplete *T. rotula*_S16 acceptor culture without bacterial inoculum served as the control. The co-culture experiments were run under the same temperature and light settings stated above and monitored daily for growth and performance. After 4 days, the *T. rotula*_S16 acceptor cultures were gravity filtered onto 3-µm polycarbonate filters and transferred into lysis buffer and stored at −20 °C. The replicated co-culture experiments (*n* = 3) were done with independently prepared bacterial inocula (Experiment I) and repeated after 6 months (Experiment II) to test if the bacterial community assembly was reproducible. In addition to Experiment I, the Experiment II included a non-diatom-derived source of the bacterial inoculum prepared from fresh seawater as well as an inoculum prepared from a fresh *T. rotula* isolate (*T. rotula*_A17).

### Bacterial DNA extraction and 16S rRNA gene sequencing

Bacterial DNA was extracted with the NucleoSpin Soil® kit (Macherey Nagel, Germany). The filter membranes obtained in Experiment I were pooled and analyzed, whereas filters of Experiment II were analyzed individually. The DNA quantity and quality were examined with a Nanodrop (Thermo Fisher Scientific, Germany). The DNA quality was verified by electrophoresis on 1% agarose gel. The axenic *T. rotula*_S16 acceptor culture was processed accordingly to verify its axenicity. Bacterial DNA was amplified using an amplicon barcoded sequencing protocol for MiSeq platforms. The V4 hypervariable region of bacterial genes was amplified using modified universal bacterial primer set 515F/806R (515F: 5′-TCGTCGGCAGCGTCAGATGTGTATAAGAGACAG GTGCCAGCMGCCGCGGTAA-3′ and 806R: 5′-GTCTCGTGGGCTCGGAGATGTGTATAAGAGACAGGGACTACHVGGGTWTCTAAT-3′). Each forward and reverse primer contained different barcode sequences with Illumina adapter overhang sequences as described previously [38, 39]. The library was prepared according to the 16S metagenomic sequencing library preparation script (https://support.illumina.com/downloads/16s_metagenomic_sequencing_library_preparation.html). 16S rRNA amplicon sequencing was subsequently performed on the Illumina MiSeq platform (Molecular Research LP; USA) following the manufacturer’s guidelines. Sequence data were deposited in the European Nucleotide Archive [40], using the data brokerage service of the German Federation for Biological Data [41], in compliance with the Minimal Information about any (X) Sequence standard [42]. They are accessible under PRJEB32927.

### Sequence data processing and bacterial community analysis

Sequencing was performed in 2 × 300-bps paired-end-mode using the MiSeq Reagent Kit v3. The trimmomatic package [43] was used to crop the 300–275 bps and a sliding window length 3 allowed an average Phred quality score of 8 to filter from 5′ to 3′ and cut when the quality dropped below the value of 8. The paired-ends were merged with Vsearch [44] with a minimum overlap of 40 bps and a maximum number of four mismatches. Sequences were reverse complemented and both directions merged into one file. The combined files were filtered for primer sequences, allowing 10% mismatch and a minimum overlap of 17 bps for the forward and 13 bps for the reverse primer. This step was followed by feature filtering to allow a maximum expected error per sequence of 1, a minimum length of 275 bps, a maximum length of 475 bps and a maximum number of ambiguities of 0. Each sample was de-replicated independently and chimera-checked de novo. All samples were pooled and de-replicated to produce a combined dataset as input for the swarm operational taxonomic unit (OTU) clustering method under default settings [45]. The most abundant amplicon of an OTU cluster was used as representative. Sequences were annotated with the default of the ribosomal database project classifier implemented in Mothur [46]. The taxonomy was assigned with the Silva v128 reference file prepared in Mothur at a confidence level of 80%. The representative annotation was used for the full OTU cluster.

### Statistical analyses

To compare community profiles between different bacterial inocula and newly established bacterial consortia on *T. rotula* in Experiments I and II, mitochondrial and chloroplast reads were excluded prior to nonparametric multivariate analyses. All reads were normalized to the median sequencing depth. Principal coordinates analysis (PCoA) was performed to determine differences between bacterial community profiles on a distance matrix using Bray–Curtis similarity measures. The differences between the grouping variables (bacterial inoculum and established *T. rotula*_S16 microbiome) and among the bacterial inocula as well as among the established *T. rotula*_S16 consortia were assessed by analysis of similarity (ANOSIM). The similarity percentage procedure was applied to identify OTUs with the highest discriminating contribution score. Statistical analyses of co-culture growth states were performed using ANOVA testing followed by post hoc, pairwise comparisons of Tukey Honest Significance Difference tests. All analyses were performed with R, version 3.4.4 (R Core Team, 2018) with Phyloseq [47] and vegan [48], and plotted with ggplot2 [49].

## Results

### Axenification and co-culture of *T. rotula_*S16 with bacteria

**Fig. 1:**
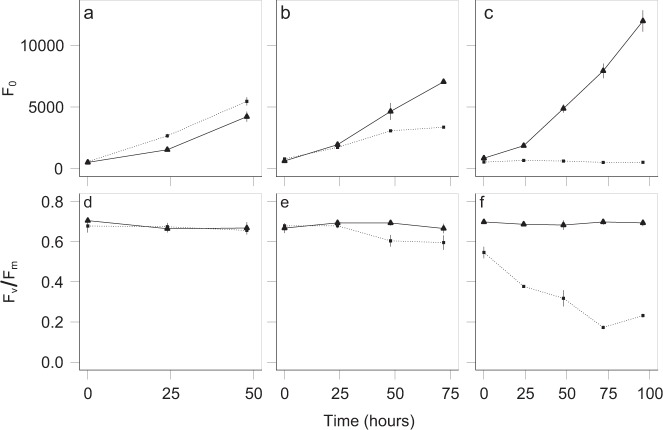
Growth and performance of *Thalassiosira rotula*_S16 in three sequential culture transfers under axenic and non-axenic conditions. Growth (*F*_0_) (**a**–**c**) and potential quantum yield (*F*_v_/*F*_m_) (**d**–**f**) of an axenic (dotted line) and non-axenic (solid line) *Thalassiosira rotula*_S16 culture under vitamin depletion for the first (**a**, **d**), second (**b**, **e**), and third (**c**, **f**) sequential transfers into ESAW^-vit^ at a starting cell density of 2000 cells mL^−1^. At the beginning of the third transfer, the culture did no longer grow (**c**) and its performance dropped below that of the vitamin-replete control (**f**). At this stage, the axenic and vitamin-deplete *T. rotula*_S16 culture was used as acceptor in co-culture experiments with bacterial inocula. Growth was determined by minimal chlorophyll fluorescence (*F*_0_) and potential quantum yield using PAM fluorometry. Each data point is the result of three culture replicates with ±1 standard deviation indicated by vertical bars.

We demonstrated that *T. rotula_*S16 critically relied on its associated bacterial community for growth and performance. This was shown with an axenic culture stripped of its supply of vitamins B_1_, B_7_, and B_12_. In comparison with the exponentially growing non-axenic and vitamin-deplete control, the axenic vitamin-deplete *T. rotula* culture ceased growth after 5–6 days. This coincided with a drop in potential quantum yield (Fig. 1) indicative of a decline in PSII, supporting the well-established paradigm that a naturally associated bacterial consortium delivers essential micronutrients for diatom growth and physiological performance [50]. At day 4, the axenic *T. rotula*_S16 culture stopped growing (Fig. 1c) and was used as recipient of the bacterial inoculum. At this stage, aliquots of the axenic, vitamin-deplete *T. rotula*_S16 culture were then inoculated and cultured with several complex bacterial consortia.

### Co-culture of axenic *T. rotula*_S16 with diverse bacterial inocula

**Fig. 2:**
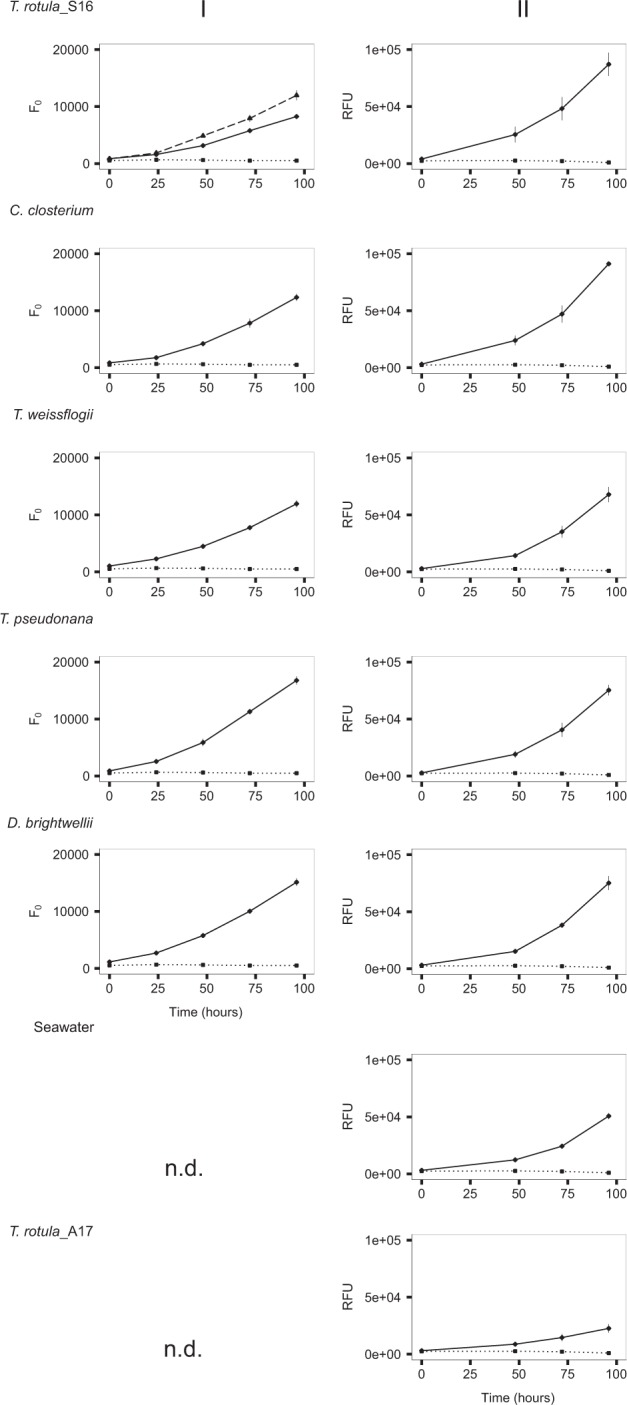
Co-culture of axenic *Thalassiosira rotula*_S16 with different bacterial inocula. Growth of *T. rotula*_S16 acceptor cultures (solid black line) in Experiments I (left column) and II (right column) inoculated with bacterial communities obtained from *T. rotula*_S16, *Cylindrotheca closterium*, *T. weissflogii*, *T. pseudonana*, *Ditylum brightwellii*, seawater, and *T. rotula*_A17 (the latter two only in Experiment II). A non-axenic vitamin-deplete *T. rotula*_S16 culture served as positive control in Experiment I (dashed line, top panel), an axenic vitamin-deplete *T. rotula*_S16 culture (dotted line) served as negative control. In Experiment I, growth was determined by minimal chlorophyll fluorescence (*F*_0_). Due to faster readings, relative fluorescence (RFU) was used as a unit of growth in Experiment II. Each data point is the result of three culture replicates with ±1 standard deviation indicated by vertical bars. n.d. (not determined).

In co-culture Experiment I, *T. rotula*_S16 inoculated with its conspecific bacterial assemblage (Fig. 2, solid line) grew equally well as the non-axenic vitamin-deplete control (Fig. 2, dashed line) and reached mid-exponential phase after 4 days. The axenic *T. rotula*_S16 culture was stagnant at this time (Fig. 2, dotted line). The *T. rotula*_S16 cultures inoculated with congeneric and heterospecific diatom bacterial assemblages in Experiments I and II grew the same or statistically better than *T. rotula*_S16 inoculated with its conspecific bacterial community (Fig. 2 and Supplementary Table S1). The only exceptions to this trend were *T. rotula*_S16 cultures inoculated with a bacterial assemblage of the conspecific autumn 2017 isolate *T. rotula*_A17 and seawater which induced significantly higher growth than the axenic control but significantly lower growth than all other inoculated cultures.

### Sequence analysis and comparison of community structures in bacterial inocula and established *T. rotula*_S16 bacterial consortia

Comparative sequence analyses of the 16S rRNA gene libraries revealed a total number of 5 million raw reads across all replicates after quality filtering (Supplementary Fig. S1). The replicates of Experiment I were pooled prior to sequencing whereas the independent replicates of Experiment II were individually sequenced. The individual bacterial inoculum communities differed significantly among each other (ANOSIM: *R* = 0.751, *p* < 0.001), whereas all established *T. rotula* bacterial consortia were statistically the same (*R* = 0.133, *p* = 0.084). This was also reflected in the clustering pattern obtained by PCoA (Fig. 4). While significant (*p* < 0.001) the differences between the community profiles of the bacterial inocula (containing 13–2071 OTUs) and the established *T. rotula* bacterial consortia (containing 11–80 OTUs, Table S2) were low as revealed by an R score of 0.154. This is likely due to the overlap of few OTUs shared across all communities, such as OTU-7, -11, -29, -30, -2, -12, and -33 (Fig. 5, panel 2). The most diverse bacterial inoculum was obtained from seawater containing 2071 OTUs. Of all bacterial OTUs in the established *T. rotula* bacterial consortia, 79.5% were Proteobacteria and 2.4% Bacteroidetes.

The different community structures of the bacterial inocula and the established *T. rotula* bacterial consortia were also apparent by PCoA (Fig. 4). There was high similarity between both conspecific bacterial inocula obtained from *T. rotula*_S16 and *T. rotula*_A17 and their corresponding established bacterial consortia. The other bacterial consortia resulting from inoculation of *T. rotula*_S16 with congeneric and heterospecific bacterial communities clustered closely with the conspecific established *T. rotula*_S16 bacterial consortia (Fig. 4).

The established bacterial consortia were characterized by 27 OTUs responsible for a cumulative abundance of 94%. The most dominant OTUs were OTU-7 (*Alteromonas* sp.), OTU-8 (unclassified), OTU-13 (*Marinomonas* sp.), and OTU-11 (*Sulfitobacter* sp.). These four OTUs alone contributed 60% to the overall reads in established *T. rotula* bacterial consortia (Fig. 5, panel 1).

When individually comparing the abundances of 27 OTUs in the inocula and established *T. rotula* bacterial consortia, three cases could be distinguished (Fig. 5, panel 2). Either an OTU was abundant in both, the inoculum as well as the established community (e.g., OTU-7). More often, OTUs were hardly abundant in the inoculum whereas abundant in the established community (e.g., OTU-8, -13, -54, -37, -49, -43, -57, and -59). Differences of highly abundant OTUs in the inoculum versus low abundance in the established community were also observed (e.g., OTU-30, -2, -12, -33, -17, and -27).

## Discussion

The outside of marine macroorganisms is commonly covered with microbial biofilms. These epibiotic microbial assemblages affect macroorganisms in various ways causing a range of positive and negative impacts [51]. While host-microbe interactions play fundamental roles in marine ecosystems, we have little understanding of the ecological processes that govern these relationships, the evolutionary processes that shape them and their synecological consequences. Cumulative evidence suggests that epibiotic microbial communities are characteristic to their living hosts [52, 53]. Yet, compared with a number of detailed microbiome studies of multicellular marine macroalgae [22, 52, 54–56], there is currently no unifying scheme or theory for bacterial community assembly on unicellular algae that underpins the divergent observations of distinct core communities on phylogenetically different microalgae to spatially and/or temporally diverse communities on conspecific microalgae [57].

To better understand the bacterial community assembly on ecologically relevant phytoplankton, we defined a model diatom that can be cultured and experimentally manipulated under controlled conditions. We chose the cosmopolitan marine diatom *T. rotula* in co-culture with bacterial communities detached from other diatoms of varying degrees of relatedness to the axenic *Thalassiosira* host or seawater. Compared with its well-studied brackish congener *T. pseudonana* [58, 59], *T. rotula* is a marine bloom-forming diatom of global abundance and significance [29, 30].

The co-cultures reached mid-exponential growth at the same time as the non-axenic control (Fig. 2 and Supplementary Table S1), suggesting that the newly established primary bacterial consortia support the same synecological function as the original microbiome of the non-axenic vitamin-deplete *T. rotula* control. Given that all diatoms used in this study are reported to have an absolute vitamin B_12_ requirement for growth [60–62], this further suggests that at least some bacteria in the different inocula (and seawater) possess vitamin synthesis capabilities. A future experimental setup resulting from these conclusions would inoculate the model system with bacterial consortia obtained from a diatom reported to lack an absolute B_12_ requirement, such as *Phaeodactylum tricornutum*, which has a flexible cobalamin demand because its genome encodes both cobalamin-dependent and independent methionine synthase [62]. In this case we would not expect to observe a growth promoting effect of an axenic, vitamin-deplete *T. rotula* culture.

### Phylogenetic composition of bacterial inoculum communities

**Fig. 3:**
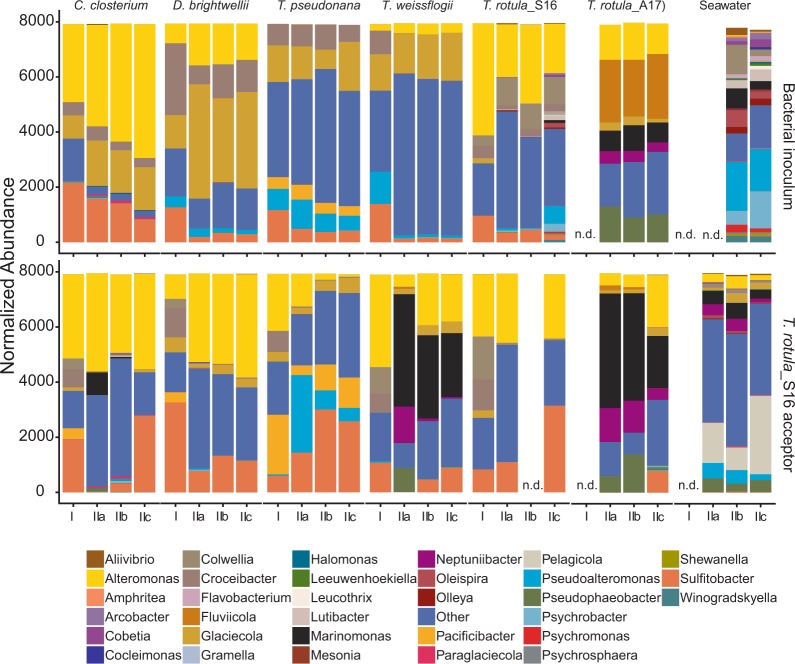
Phylogenetic composition of established *T. rotula*_S16 bacterial consortia (bottom row) and bacterial inoculum communities detached from different diatoms and seawater (top row). The stacked bar plots show the normalized abundance of OTUs at the genus level. The pooled replicates of Experiment I are denoted as (I). Individual replicates (a, b, c) of Experiment II are denoted as (IIa–c). n.d. (not determined).

**Fig. 4:**
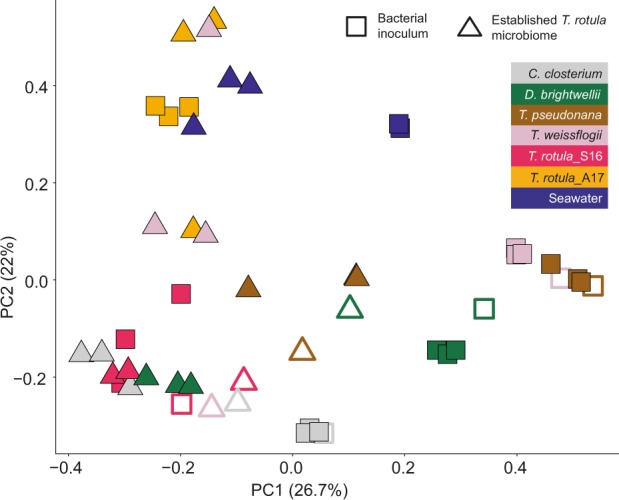
Principle coordinate analysis (PCoA) of bacterial community compositions in established *Thalassiosira rotula*_S16 bacterial consortia (triangles) and bacterial inocula (squares) obtained from non-axenic *T. rotula*_S16, *T. rotula*_A17, *T. weissflogii*, *T. pseudonana*, *Ditylum brightwellii*, *Cylindrotheca closterium*, and seawater. Treatments of Experiment I and II are denoted by open (I) and closed (II) symbols, respectively.

In support of recent studies, the phylogenetic composition of associated bacterial consortia among the various diatoms differed significantly [2, 18, 19, 63]. The consortia were dominated by Alteromonadaceae and Rhodobacteraceae, which is in agreement with prior studies [2, 19]. The phylogenetically most diverse bacterial community was obtained from seawater, which is consistent with studies comparing free-living and particle-associated bacteria (including those associated with algae) [50, 64]. The repeated sequence analyses of bacterial consortia on diatoms sampled 6 months apart were highly reproducible (Figs. 3 and 4). Such strong conservation across strains cultivated from different seasons is in agreement with recent studies [16]. The *T. rotula* isolates obtained in spring 2016 and autumn 2017 revealed different bacterial consortia, with the autumn isolate rich in Cryomorphaceae and Oceanospirillaceae while lacking Colwelliaceae (Fig. 3). Similar observations of the influence of season on the bacterial community structure have been made with other conspecific diatoms [21].

### Phylogenetic composition of established *T. rotula* bacterial consortia

The bacteria in the different inocula were presumably adapted to a diatom-associated life style, yet the newly established *T. rotula*_S16 bacterial consortia consisting of 11–80 OTUs were statistically different from these source communities (Fig. 4). Notably, the established *T. rotula* bacterial consortia were compositionally and quantitatively most similar to each of their corresponding conspecific source community (i.e., *T. rotula* strains S16 and A17), thus validating the experimental approach of effectively dissociating and re-establishing their diatom-specific bacterial consortia (Fig. 4).

Overall, the co-culture experiment revealed a convergent assembly of highly diverse and compositionally different bacterial communities (consisting of 13–2071 OTUs) towards a rather defined and reduced *T. rotula* core community. Even the most diverse community, seawater, containing 2071 OTUs, was reduced to this core community, dominated by Rhodobacteraceae (30.5%), Alteromonadaceae (27.7%), and Oceanospirillales (18.5%) (Figs. 3 and 4).

**Fig. 5:**
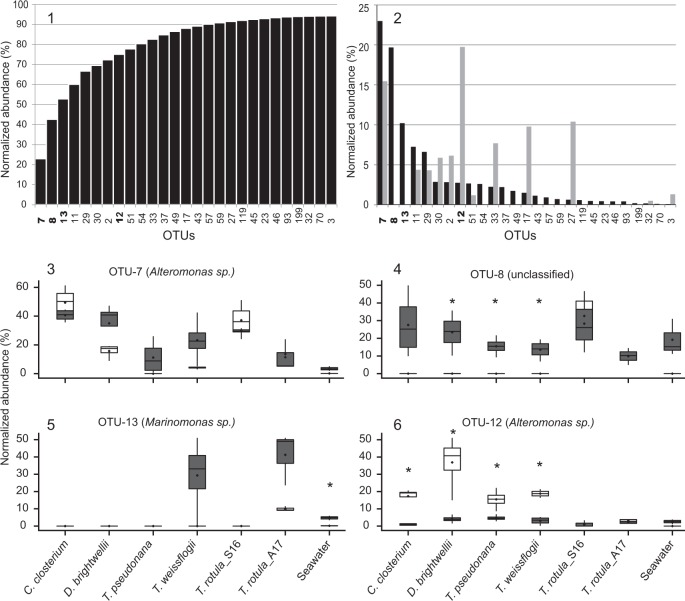
Abundance and contribution of OTUs to the *Thalassiosira rotula* core microbiome. **Panel 1** Cumulative normalized abundance of 27 OTUs comprising 94% of the established *T. rotula* bacterial consortia (bold OTU numbers are presented in panel 3–6). **Panel 2** Comparison of individual OTU abundances averaged across all inocula (gray bars) and established acceptor bacterial consortia (black bars). **Panel 3–6** Normalized abundance of OTU-7, -8, -13, and -12 in individual bacterial inocula (white box plots) and corresponding established *T. rotula* bacterial consortia (gray box plots). Significant differences in OTU abundances between bacterial inocula and established consortia are marked with an asterisk.

The differences in relative abundance of certain OTUs in the established bacterial consortia compared with the inoculum were either the same (indicating neutral uptake), significantly higher (indicating favorable uptake), or significantly lower (indicating disadvantageous uptake) (Fig. 5, panel 2–6). The most drastic qualitative and quantitative transition from the inoculum to the core community was observed in the co-culture setup of a seawater bacterial community with the axenic *T. rotula* culture, supporting a parallel study using seawater bacterioplankton as inoculum with axenic *T. rotula* [65].

Our experimental setup was based on the hypothesis that all bacteria in the different inocula (except for the seawater community) possessed the genetic and metabolic abilities to colonize the axenic *T. rotula* surface and be sustained on diatom-derived organic carbon. The strong phylogenetic convergence and high degree of overlap among comparatively few OTUs shared across the established *T. rotula* bacterial consortia was instead indicative of a highly selected, stable, and reproducible core bacterial community. This conclusion was further supported by the diverse seawater bacterial community converging to statistically the same characteristic community structure (Figs. 3 and 4). Remarkably, despite high statistical similarity among all established *T. rotula_*S16 bacterial consortia, the *T. rotula_*S16 cultures inoculated with bacteria obtained from *T. rotula*_A17 and seawater, both sampled in autumn 2017, revealed significantly lower growth than the other co-cultures (Fig. 2). Since both of these established consortia clustered closely by PCoA (Fig. 4), these results suggest that even small differences in the overall community composition can significantly affect the diatom host performance.

Together, these data reject the hypothesis that the bacterial consortium assembly on *T. rotula* is due to stochastic recruitment according to the lottery hypothesis. Instead, bacterial colonization of the diatom results from a reproducible selection pattern of individual OTUs from the inoculum into the corresponding established consortia. This clearly suggests a steering force or “habitat filtering effect” [66]. The host diatom is likely to drive the initial filtering or selection, providing nutritional conditions that promote the growth of certain microbes over others. Indeed, the low-molecular weight sugars and amino acids released by diatoms are the chemical cues that allow flagellated and chemotactic bacterial groups such as Rhodobacteraceae, Alteromonadaceae, and Oceanospirillales to be enriched within the phycosphere and potentially interact with the HMW substratum (e.g., polysaccharides) to which biofilm cells adhere. It is moreover possible that host traits act in tandem with feedback loops between the host and microbes, e.g., via the release of antibacterial compounds [67, 68], as well as among microbes [65, 69], allowing the establishment of a predictable assemblage of OTUs in the community.

In our study, the 4-day-old primary *T. rotula* bacterial consortium was compositionally highly similar to the inoculum sourced from an established, several month-old donor culture, suggesting that the bacterial core community indeed represents a long-term stable-state system. Our findings are in contrast to a recent comparative study [65] demonstrating a shift in the *T. rotula* associated bacterial composition over a time course of 8 days, possibly due to changes in the host physiology (e.g., growth and metabolism).

Cumulatively, the co-culture experiment used in this study offers experimental evidence that initial bacterial epibiosis of the marine model diatom *T. rotula* is characteristically underpinned by the niche-based diatom microscale environment and bacterial species-specific metabolic characteristics resulting in a remarkably stable and reproducible bacterial core community. This knowledge in tandem with further investigations of the role and function of stable core bacterial members is crucial when studying the performance and resilience of the diatom holobiont in the context of environmental change. The feedback between the marine carbon cycle and ocean warming make such dynamic hotspots for the microbial loop critical interactions to consider in light of our rapidly impacted oceans.

## Supplementary information

SUPPLEMENTAL MATERIAL
