# Supplementary material for: Niche-based assembly of bacterial consortia on the diatom Thalassiosira rotula is stable and reproducible
Source: ISME J. 2020 Mar 23;14(6):1614–25. doi: 10.1038/s41396-020-0631-5 (PMC7242391; doi:10.1038/s41396-020-0631-5)
Supplement: Supplementary file 1 — SUPPLEMENTAL MATERIAL [file 41396_2020_631_MOESM1_ESM.docx]

**Supplementary section**

**Sequence analysis of *Thalassiosira rotula***

Primer sequences:

EUKA-F SSU: 5'-AACCTGGTTGATCCTGCCAGT-3' (Medlin et al., 1988)

EUKB-R SSU: 5'-TGATCCTTCTGCAGGTTCACCTAC-3' (Medlin et al., 1988)

LSU D1R-F LSU: 5'-ACCCGCTGAATTTAAGCATA-3' (Scholin et al., 1994)

LSU D2C-R LSU: 5'-CCTTGGTCCGTGTTTCAAGA-3' (Scholin et al., 1994)

Sequence and BLAST results:

***T. rotula*_A17, LSU D1R-F LSU**

| Blast: *Thalassiosira rotula* 28S rRNA gene, strain thal. rot | , 99.50% |  |
| --- | --- | --- |

AAGAATAACAGGATTCCCCTAGTAACGGCGAGTGAAGCGGGAAGAGCTCACCAGTGAATCTGTGTAACCGGAAACGGTGCACCGAATTGTGGTCTGGAGAAGTACTGTCGGCCGTGTTCCCGGGCCAAGTCTCTTGGAAAAGGGCAGCTGAGAGGGTGAAACTCCCGTTCTTGCCTGGGAACATTGTGCTTTGGCATATACTTTCTACGAGTCGAGTTGCTTGGGATTGCAGCTCAAATTTGGTGGTAAATTCCATCTAAAGCTAAATATTGGTGGGATACCGATAGTGCACAAGTACCGTGAGGGAAAGATGCAAAGAACTTTGAAAAGAGAGTTAAAGAGTACCTGAAATTGTTGAAAGGGAAGCGAAGGAAACCAGTGCCGAAGCTTAGTCATACTTCTCTTACTACTTGTGGTAAGGGCGCTGTGGCTTTGCGTGGGTCAGCATCGGCTCTTTGCCTGGGATATATCTTCAGTAGGTAGACGACCTCTTCGGAGGTGAGTGCCTATTGTTGCTATTCCGGGTTGGGCTGAGGTCAGTCACTCTGTGCTCGTGATGCTGGCAAAATGGTTTTCTTTACCCCGTCTTGAAACACGGGACCAAGGT

***T. rotula*_A17, LSU D2C-R LSU**

| Blast: *Thalassiosira rotula* 28S ribosomal RNA gene, partial sequence | , 99.33% |
| --- | --- |

GGGGAAATTTTGCAGCATCACGAGCACAGAGTGACTGACCTCAGCCCAACCCGGAATAGCAACAATAGGCACTCACCTCCGAAGAGGTCGTCTACCTACTGAAGATATATCCCAGGCAAAGAGCCGATGCTGACCCACGCAAAGCCACAGCGCCCTTACCACAAGTAGTAAGAGAAGTATGACTAAGCTTCGGCACTGGTTTCCTTCGCTTCCCTTTCAACAATTTCAGGTACTCTTTAACTCTCTTTTCAAAGTTCTTTGCATCTTTCCCTCACGGTACTTGTGCACTATCGGTATCCCACCAATATTTAGCTTTAGATGGAATTTACCACCAAATTTGAGCTGCAATCCCAAGCAACTCGACTCGTAGAAAGTATATGCCAAAGCACAATGTTCCCAGGCAAGAACGGGAGTTTCACCCTCTCAGCTGCCCTTTTCCAAGAGACTTGGCCCGGGAACACGGCCGACAGTACTTCTCCAGACCACAATTCGGTGCACCGTTTCCGGTTACACAGATTCACATGGTGAGCTCTTCCCGCTTCACTCGCCGTTACTAGGGGAATCCTTGTTAGTTTCTTTTCCTCCGCTTAATTATATGCTTTTTT

***T. rotula*_A17, EUKA-F SSU**

| Blast: *Thalassiosira rotula* strain CCMP1812 18S small subunit ribosomal RNA gene, partial sequence, 99.92% |  |
| --- | --- |

CCCAGGATTAAGCCATGCATGTCTAAGTATAACTCTTTTACTTTGAAAACTGCGAACGGCTCATTATATCAGTTATAGTTTATTTGATAGTCCCTTACTACTTGGATACCCGTAGTAATTCTAGAGCTAATACATGCATCAATACCCAACTGTTCGCGGAAGGGTAGTATTTATTAGGTATAGACCAACCCTCTTCGGAGGTGCTTTGGTGATTCATAATAACTTTTCGAATCGCATGGCTCCATGCCGGCGATGGATCATTCAAGTTTCTGCCCTATCAGCTTTGGATGGTAGTGTATTGGACTACCATGGCTTTAACGGGTAACGAATTGTTAGGGCAAGATTTCGGAGAGGGAGCCTGAGAGACGGCTACCACATCCAAGGAAGGCAGCAGGCGCGTAAATTACCCAATCCTGACACAGGGAGGTAGTGACAATAAATAACAATGCCGGGCCTTTACAGGTCTGGCAATTGGAATGAGAACAATTTAAATCCCTTATCGAGTATCAATTGGAGGGCAAGTCTGGTGCCAGCAGCCGCGGTAATTCCAGCTCCAATAGCGTATATTAAAGTTGTTGCAGTTAAAAAGCTCGTAGTTGGATTTCTGGCAGGAGTGACCGGTCACGCACTCTGTGCGTGAACTTGTGTTGTCTCTGGCCATCCTTGGGGAGATCCTGTTTGGCATTAAGTTGTCGGGCAGGGGATACCCATCGTTTACTGTGAAAAAATTAGAGTGTTTAAAGCAGGCTTATGCCGTTGAATATATTAGCATGGAATAATAAGATAGGACTTCGGAACTATTTTGTTGGTTTGCGTTACGAAGTAATGATTAATAGGGACAGTTGGGGGTATTCGTATTTCGTTGTCAGAGGTGAAATTCTTGGATTTCCGAAAGACGAACTACTGCGAAAGCATTTACCAAGGATGTTTTCATTAATCAAGAACGAAAGTTAGGGGATCGAAGATGATTAGATACCATCGTAGTCTTAACCATAAACTATGCCGACTCGGGATCGGAGGTTGTTTTTTGACTCCTTCGGCACCGTATGAGAAATCAAAGTCTTTGGGTTCCGGGGGGGAGTATGGTCGCAAGGCTGAAACTTAAAGAAATTGACGGAAGGGCACCACCAGGAGTGGAACCTGCGGCTTAATTTGACTCAACACGGGAAAACTTACCAGGTCCAGACATA

***T. rotula*_A17, EUKB-R SSU**

| Blast: *Thalassiosira rotula* 18S ribosomal RNA gene, partial sequence | , 98.20% |
| --- | --- |

TGTTCGACTTCACCTTCCTCTAATGATAAGGTTCGGACAGTTCTCGCGGTCAGGCCCCAATGAAGGAGCCAAGCCACAATCCCGAGTCCTCACCGGACCATTCAATCGGTAGGTGCGACGGGCGGTGTGTACAAAGGGCAGGGACGTAATCATTGCGGTTTGATGAACCGCGATTACTAGGAATTCCTCGTTCAAGATTAATAATTGCAATAATCTATCCCTATCACGATGCAAGTTAACAAGATTACCCAGGCCTCTCGGCCAAGGTTATATGCTCGTTGAGTGCATCAGTGTAACGCGCGTGCGGCCCAGGACATCAAAGGGCATCACAGACCTGTTATTGCCGCCATCTTCCTTCATCTTGTAGAATGAACGTCCCTCTAAGAAGCTCTTAACCAATCAAAAACGATTAGTAGAACTATTTAGCAGGCGGCGGTCTCGTTCGTTAACGGAATTAACCAGACAAATCACTCCACCAACTAAGAACGGCCATGCACCACCACCCATAGAATCAAGAAAGAACTCTCAATCTGTCAATCCTCACTATGTCTGGACCTGGTAAGTTTTCCCGTGTTGAGTCAAATTAAGCCGCAGGTTCCACTCCTGGTGGTGCCCTTCCGTCAATTTCTTTAAGTTTCAGCCTTGCGACCATACTCCCCCCGGAACCCAAAGACTTTGATTTCTCATACGGTGCCGAAGGAGTCAAAAAACAACCTCCGATCCCGAGTCGGCATAGTTTATGGTTAAGACTACGATGGTATCTAATCATCTTCGATCCCCTAACTTTCGTTCTTGATTAATGAAAACATCCTTGGTAAATGCTTTCGCAGTAGTTCGTCTTTCGGAAATCCAAGAATTTCACCTCTGACAACGAAATACGAATACCCCCAACTGTCCCTATTAATCATTACTTCGTAACGCAAACCAACAAAATAGTTCCGAAGTCCTATCTTATTATTCCATGCTAATATATTCAACGGCATAAGCCTGCTTTAAACACTCTAATTTTTTCACAGTAAACGATGGGTATCCCCTGCCCGACAACTTAATGCCAAACAGGATCTCCCCAAGGATGGCCAGAGACAACACAAGTTCACGCACAGAGTGCGTGACCGGTCACTCCTGCCAAAATCCACTACGAGCTTTTAACTGCACAACTTTAATAACGCTATTGGAACTGGAATACCGCGCTGCTGGCCCAGATTGCCTCCATTGAAATCGAAAGGGATTAATTGTTCCATTCCATTGCCAACC

***T. rotula*_S16, EUKA-F SSU**

| Blast: *Thalassiosira rotula* strain CCMP1812 18S small subunit ribosomal RNA gene, partial sequence, 99.82% |  |
| --- | --- |

AGAATTAAGCCATGCATGTCTAAGTATAACTCTTTTACTTTGAAACTGCGAACGGCTCATTATATCAGTTATAGTTTATTTGATAGTCCCTTACTACTTGGATACCCGTAGTAATTCTAGAGCTAATACATGCATCAATACCCAACTGTTCGCGGAAGGGTAGTATTTATTAGGTATAGACCAACCCTCTTCGGAGGTGCTTTGGTGATTCATAATAACTTTTCGAATCGCATGGCTCCATGCCGGCGATGGATCATTCAAGTTTCTGCCCTATCAGCTTTGGATGGTAGTGTATTGGACTACCATGGCTTTAACGGGTAACGAATTGTTAGGGCAAGATTTCGGAGAGGGAGCCTGAGAGACGGCTACCACATCCAAGGAAGGCAGCAGGCGCGTAAATTACCCAATCCTGACACAGGGAGGTAGTGACAATAAATAACAATGCCGGGCCTTTACAGGTCTGGCAATTGGAATGAGAACAATTTAAATCCCTTATCGAGTATCAATTGGAGGGCAAGTCTGGTGCCAGCAGCCGCGGTAATTCCAGCTCCAATAGCGTATATTAAAGTTGTTGCAGTTAAAAAGCTCGTAGTTGGATTTCTGGCAGGAGTGACCGGTCACGCACTCTGTGCGTGAACTTGTGTTGTCTCTGGCCATCCTTGGGGAGATCCTGTTTGGCATTAAGTTGTCGGGCAGGGGATACCCATCGTTTACTGTGAAAAAATTAGAGTGTTTAAAGCAGGCTTATGCCGTTGAATATATTAGCATGGAATAATAAGATAGGACTTCGGAACTATTTTGTTGGTTTGCGTTACGAAGTAATGATTAATAGGGACAGTTGGGGGTATTCGTATTTCGTTGTCAGAGGTGAAATTCTTGGATTTCCGAAAGACGAACTACTGCGAAAGCATTTACCAAGGATGTTTTCATTAATCAAGAACGAAAGTTAGGGGATCGAAGATGATTAGATACCATCGTAGTCTTAACCATAAACTATGCCGACTCGGGATCGGAGGTTGTTTTTTGACTCCTTCGGCACCGTATGAGAAATCAAAGTCTTTGGGTTCCGGGGGAGTATGGTCGCAAGGCTGAAACTTAAAGAAATTGACGGAAGGGCACCACCAG

***T. rotula*_S16, EUKB-R SSU**

| Blast: *Thalassiosira rotula* 18S ribosomal RNA gene, partial sequence | 99.07% |
| --- | --- |

GGTTAGACTTCACCTTCCTCTAATGATAAGGTTCGGACAAGTTCTCGCGGTCAGGCCCCAATGAAGGAGCCAAGCCACAATCCCGAGTCCTCACCGGACCATTCAATCGGTAGGTGCGACGGGCGGTGTGTACAAAGGGCAGGGACGTAATCATTGCGGTTTGATGAACCGCGATTACTAGGAATTCCTCGTTCAAGATTAATAATTGCAATAATCTATCCCTATCACGATGCAAGTTAACAAGATTACCCAGGCCTCTCGGCCAAGGTTATATGCTCGTTGAGTGCATCAGTGTAACGCGCGTGCGGCCCAGGACATCAAAGGGCATCACAGACCTGTTATTGCCGCCATCTTCCTTCATCTTGTAGAATGAACGTCCCTCTAAGAAGCTCTTAACCAATCAAAAACGATTAGTAGAACTATTTAGCAGGCGGCGGTCTCGTTCGTTAACGGAATTAACCAGACAAATCACTCCACCAACTAAGAACGGCCATGCACCACCACCCATAGAATCAAGAAAGAACTCTCAATCTGTCAATCCTCACTATGTCTGGACCTGGTAAGTTTTCCCGTGTTGAGTCAAATTAAGCCGCAGGTTCCACTCCTGGTGGTGCCCTTCCGTCAATTTCTTTAAGTTTCAGCCTTGCGACCATACTCCCCCCGGAACCCAAAGACTTTGATTTCTCATACGGTGCCGAAGGAGTCAGAAAACAACCTCCGATCCCGAGTCGGCATAGTTTATGGTTAAGACTACGATGGTATCTAATCATCTTCGATCCCCTAACTTTCGTTCTTGAGTAATGAGAACATCCTTGGTAAATGCTTTCGCAGAAGTTCGACTTTCGGAAATCGAAG

**T. rotula_S16, LSU D1R-F LSU**

| Blast: *Thalassiosira rotula* 28S rRNA gene, strain thal.rot | , 99.83% |
| --- | --- |

GAGAAGAACTAACAAGGATTCCCCTAGTAACGGCGAGTGAAGCGGGAAGAGCTCACCATGTGAATCTGTGTAACCGGAAACGGTGCACCGAATTGTGGTCTGGAGAAGTACTGTCGGCCGTGTTCCCGGGCCAAGTCTCTTGGAAAAGGGCAGCTGAGAGGGTGAAACTCCCGTTCTTGCCTGGGAACATTGTGCTTTGGCATATACTTTCTACGAGTCGAGTTGCTTGGGATTGCAGCTCAAATTTGGTGGTAAATTCCATCTAAAGCTAAATATTGGTGGGATACCGATAGTGCACAAGTACCGTGAGGGAAAGATGCAAAGAACTTTGAAAAGAGAGTTAAAGAGTACCTGAAATTGTTGAAAGGGAAGCGAAGGAAACCAGTGCCGAAGCTTAGTCATACTTCTCTTACTACTTGTGGTAAGGGCGCTGTGGCTTTGCGTGGGTCAGCATCGGCTCTTTGCCTGGGATATATCTTCAGTAGGTAGACGACCTCTTCGGAGGTGAGTGCCTATTGTTGCTATTCCGGGTTGGGCTGAGGTCAGTCACTCTGTGCTCGTGATGCTGGCAAAATGGTTTTCTTTACCCCGTCTTGAACGGGGA

***T. rotula*_S16, LSU *D2C-R LSU***

| Blast: *Thalassiosira rotula* 28S ribosomal RNA gene, partial sequence | , 99.18% |
| --- | --- |

GGGGAAATTTTGCAGCATCACGAGCACAGAGTGACTGACCTCAGCCCAACCCGGAATAGCAACAATAGGCACTCACCTCCGAAGAGGTCGTCTACCTACTGAAGATATATCCCAGGCAAAGAGCCGATGCTGACCCACGCAAAGCCACAGCGCCCTTACCACAAGTAGTAAGAGAAGTATGACTAAGCTTCGGCACTGGTTTCCTTCGCTTCCCTTTCAACAATTTCAGGTACTCTTTAACTCTCTTTTCAAAGTTCTTTGCATCTTTCCCTCACGGTACTTGTGCACTATCGGTATCCCACCAATATTTAGCTTTAGATGGAATTTACCACCAAATTTGAGCTGCAATCCCAAGCAACTCGACTCGTAGAAAGTATATGCCAAAGCACAATGTTCCCAGGCAAGAACGGGAGTTTCACCCTCTCAGCTGCCCTTTTCCAAGAGACTTGGCCCGGGAACACGGCCGACAGTACTTCTCCAGACCACAATTCGGTGCACCGTTTCCGGTTACACAGATTCACATGGTGAGCTCTTCCCGCTTCACTCGCCGTTACTAGGGGAATCCTTGTTAGTTTCTTTTCCTCCGCTTAATTATATGCTTAAATTTCAGCGGGTAT

***T. rotula*_S16 axenic, EUKA-F SSU**

| Blast: *Thalassiosira rotula* strain CCMP1812 18S small subunit ribosomal RNA gene, partial sequence, 99.16% |  |
| --- | --- |

CTCCAAGAATTAAGCCATGCATGTCTAAGTATAACTCTTTTACTTTGAAAACTGCGAACGGCTCATTATATCAGTTATAGTTTATTTGATAGTCCCTTACTACTTGGATACCCGTAGTAATTCTAGAGCTAATACATGCATCAATACCCAACTGTTCGCGGAAGGGTAGTATTTATTAGGTATAGACCAACCCTCTTCGGAGGTGCTTTGGTGATTCATAATAACTTTTCGAATCGCATGGCTCCATGCCGGCGATGGATCATTCAAGTTTCTGCCCTATCAGCTTTGGATGGTAGTGTATTGGACTACCATGGCTTTAACGGGTAACGAATTGTTAGGGCAAGATTTCGGAGAGGGAGCCTGAGAGACGGCTACCACATCCAAGGAAGGCAGCAGGCGCGTAAATTACCCAATCCTGACACAGGGAGGTAGTGACAATAAATAACAATGCCGGGCCTTTACAGGTCTGGCAATTGGAATGAGAACAATTTAAATCCCTTATCGAGTATCAATTGGAGGGCAAGTCTGGTGCCAGCAGCCGCGGTAATTCCAGCTCCAATAGCGTATATTAAAGTTGTTGCAGTTAAAAAGCTCTAGTTGGATTTCTGGCAGGAGTGACCGGTCACGCACTCTGTGCGTGAACTTGTGTTGTCTCTGGCCATCCTTGGGGAGATCCTGTTTGGCATTAAGTTGTCGGGCAGGGGATACCCATCGTTTACTGTGAAAAAATTAGAGTGTTTAAAGCAGGCTTATGCCGTTGAATATATTAGCATGGAATAATAAGATAGGACTTCGGAACTATTTTGTTGGTTTGCGTTACGAAGTAATGATTAATAGGGACAGTTGGGGGTATTCGTATTTCGTTGTCAGAGGTGAAATTCTTGGATTTCCGAAAGACGAACTACTGCGAAAGCATTTACCAAGGATGTTTTCATTAATCAAGAACGAAAGTTAGGGGATCGAAGATGATTAGATACCATCGTAGTCTTAACCATAAACTATGCCGACTCGGGATCGGAGGTTGTTTTTTGACTCCTTCGGCACCGTATGAGAAATCAAAGTCTTTGGGTTCCGGGGGGAGTATGGTCGCAAGGCTGAAACTTAAAGAAATTGACGGAAGGGCACACCAGGAGTGAACCTGCGGCTTATTTGACTCACACGGAAAACTTACAGGTCCAAACTAGT

***T. rotula*_S16 axenic, EUKB-R SSU**

| Blast: *Thalassiosira rotula* 18S ribosomal RNA gene, partial sequence | , 99.59% |
| --- | --- |

GGTTCGACTTCACCTTCCTCTAATGATAAGGTTCGGACAAGTTCTCGCGGTCAGGCCCCAATGAAGGAGCCAAGCCACAATCCCGAGTCCTCACCGGACCATTCAATCGGTAGGTGCGACGGGCGGTGTGTACAAAGGGCAGGGACGTAATCATTGCGGTTTGATGAACCGCGATTACTAGGAATTCCTCGTTCAAGATTAATAATTGCAATAATCTATCCCTATCACGATGCAAGTTAACAAGATTACCCAGGCCTCTCGGCCAAGGTTATATGCTCGTTGAGTGCATCAGTGTAACGCGCGTGCGGCCCAGGACATCAAAGGGCATCACAGACCTGTTATTGCCGCCATCTTCCTTCATCTTGTAGAATGAACGTCCCTCTAAGAAGCTCTTAACCAATCAAAAACGATTAGTAGAACTATTTAGCAGGCGGCGGTCTCGTTCGTTAACGGAATTAACCAGACAAATCACTCCACCAACTAAGAACGGCCATGCACCACCACCCATAGAATCAAGAAAGAACTCTCAATCTGTCAATCCTCACTATGTCTGGACCTGGTAAGTTTTCCCGTGTTGAGTCAAATTAAGCCGCAGGTTCCACTCCTGGTGGTGCCCTTCCGTCAATTTCTTTAAGTTTCAGCCTTGCGACCATACTCCCCCCGGAACCCAAAGACTTTGATTTCTCATACGGTGCCGAAGGAGTCAAAAAACAACCTCCGATCCCGAGTCGGCATAGTTTATGGTTAAGACTACGATGGTATCTAATCATCTTCGATCCCCTAACTTTCGTTCTTGATTAATGAAAACATCCTTGGTAAATGCTTTCGCAGTAGTTCGTCTTTCGGAAATCCAAGAATTTCACCTCTGACAACGAAATACGAATACCCCCAACTGTCCCTATTAATCATTACTTCGTAACGCAAACCAACAAAATAGTTCCGAAGTCCTATCTTATTAGTCCATGCTAATATATTCCACGGG

***T. rotula*_S16 axenic, LSU D1R-F LSU**

| Blast: *Thalassiosira rotula* 28S rRNA gene, strain thal.rot, | 99.66% |
| --- | --- |

AGAAGAATAACAGGGATTCCCCTAGTAACGGCGAGTGAAGCGGGAAGAGCTCACCATGTGAATCTGTGTAACCGGAAACGGTGCACCGAATTGTGGTCTGGAGAAGTACTGTCGGCCGTGTTCCCGGGCCAAGTCTCTTGGAAAAGGGCAGCTGAGAGGGTGAAACTCCCGTTCTTGCCTGGGAACATTGTGCTTTGGCATATACTTTCTACGAGTCGAGTTGCTTGGGATTGCAGCTCAAATTTGGTGGTAAATTCCATCTAAAGCTAAATATTGGTGGGATACCGATAGTGCACAAGTACCGTGAGGGAAAGATGCAAAGAACTTTGAAAAGAGAGTTAAAGAGTACCTGAAATTGTTGAAAGGGAAGCGAAGGAAACCAGTGCCGAAGCTTAGTCATACTTCTCTTACTACTTGTGGTAAGGGCGCTGTGGCTTTGCGTGGGTCAGCATCGGCTCTTTGCCTGGGATATATCTTCAGTAGGTAGACGACCTCTTCGGAGGTGAGTGCCTATTGTTGCTATTCCGGGTTGGGCTGAGGTCAGTCACTCTGTGCTCGTGATGCTGGCAAAATGGTTTTCTTTACCCCGTCTTGACGGGG

***T. rotula*_S16 axenic, LSU D2C-R LSU**

| Blast: *Thalassiosira rotula* 28S ribosomal RNA gene, partial sequence, | 99.49% |
| --- | --- |

TGGGAAATTTTGCCAGCATCACGAGCACAGAGTGACTGACCTCAGCCCAACCCGGAATAGCAACAATAGGCACTCACCTCCGAAGAGGTCGTCTACCTACTGAAGATATATCCCAGGCAAAGAGCCGATGCTGACCCACGCAAAGCCACAGCGCCCTTACCACAAGTAGTAAGAGAAGTATGACTAAGCTTCGGCACTGGTTTCCTTCGCTTCCCTTTCAACAATTTCAGGTACTCTTTAACTCTCTTTTCAAAGTTCTTTGCATCTTTCCCTCACGGTACTTGTGCACTATCGGTATCCCACCAATATTTAGCTTTAGATGGAATTTACCACCAAATTTGAGCTGCAATCCCAAGCAACTCGACTCGTAGAAAGTATATGCCAAAGCACAATGTTCCCAGGCAAGAACGGGAGTTTCACCCTCTCAGCTGCCCTTTTCCAAGAGACTTGGCCCGGGAACACGGCCGACAGTACTTCTCCAGACCACAATTCGGTGCACCGTTTCCGGTTACACAGATTCACATGGTGAGCTCTTCCCGCTTCACTCGCCGTTACTAGGGGAATCCTTGTTAGTTTCTTTTCCTCCGCTTAATTATATGA

References:

Medlin L, Elwood HJ, Stickel S, Sogin ML. The characterization of enzymatically amplified eukaryotic 16S-like rRNA-coding regions. Gene. 1988;71:491-99.

Scholin CA, Herzog M, Sogin M, Anderson DM. Identification of group- and strain-specific genetic markers for globally distributed Alexandrium (Dinophaceae). II. Sequence analysis of the fragment of the LSU rRNA gene. J Phycol. 1994;30:999-1011.

Table S1: Pairwise growth comparison (Tukey HSD test) of *T. rotula* (S16) acceptor cultures at day 4 with conspecific versus congeneric or heterospecific diatom bacterial consortia. The symbols (+), (=) and (-) denote (better), (equal) or (lower) diatom abundance in acceptor cultures with congeneric or heterospecific compared to conspecific bacterial inocula at day 4.

| Bacterial inoculum obtained from: | **Experiment I** | **Experiment II** |
| --- | --- | --- |
|  | *T. rotula*_S16  with conspecific microbiome | *T. rotula*_S16  with conspecific microbiome |
| *C. closterium* | **+** (p < 0.001) | **=** (p = 0.992) |
| *T. weissflogii* | **+** (p < 0.001) | **=** (p = 0.067) |
| *T. pseudonana* | **+** (p < 0.001) | **=** (p = 0.464) |
| *D. brightwellii* | **+** (p < 0.001) | **=** (p = 0.445) |
| Seawater | n.d. | **-** (p < 0.001) |
| *T. rotula*_A17 | n.d. | **-** (p < 0.005) |

Table S2: Numbers of total reads and OTUs (minimum of 3 reads) in bacterial inocula and established *T. rotula*_S16 bacterial consortia in Experiments I and II a-c; n.d. (not determined)

| **Bacterial inoculum source** | **Replicate** | **total # reads** | **# OTUs** | **Established *T. rotula*_S16 microbiomes** | **Replicate** | **total # reads** | **# OTUs** |
| --- | --- | --- | --- | --- | --- | --- | --- |
| *T. rotula_*S16 | I | 9101 | 30 | *T. rotula_*S16 | I | 7665 | 34 |
|  | II a | 2890 | 23 |  | II a | 4222 | 17 |
|  | II b | 3136 | 19 |  | II b | n.d. | n.d. |
|  | II c | 3910 | 58 |  | II c | 77359 | 77 |
| *T. rotula*_A17 | I | n.d. | n.d. | *T. rotula*_A17 | I | n.d. | n.d. |
|  | II a | 18302 | 43 |  | II a | 13737 | 47 |
|  | II b | 13249 | 42 |  | II b | 25014 | 45 |
|  | II c | 10619 | 34 |  | II c | 6944 | 35 |
| *T. pseudonana* | I | 25061 | 68 | *T. pseudonana* | I | 10885 | 38 |
|  | II a | 18683 | 49 |  | II a | 4164 | 11 |
|  | II b | 19994 | 48 |  | II b | 4420 | 16 |
|  | II c | 21273 | 57 |  | II c | 5572 | 18 |
| *T. weissflogii* | I | 5916 | 29 | *T. weissflogii* | I | 16463 | 51 |
|  | II a | 11908 | 30 |  | II a | 23653 | 66 |
|  | II b | 11068 | 32 |  | II b | 10846 | 29 |
|  | II c | 10400 | 31 |  | II c | 7574 | 32 |
| *D. brightwellii* | I | 2062 | 19 | *D. brightwellii* | I | 6260 | 31 |
|  | II a | 1284 | 22 |  | II a | 3478 | 24 |
|  | II b | 911 | 13 |  | II b | 4534 | 18 |
|  | II c | 2808 | 32 |  | II c | 8075 | 27 |
| *C. closterium* | I | 2486 | 24 | *C. closterium* | I | 6109 | 26 |
|  | II a | 3761 | 22 |  | II a | 5178 | 16 |
|  | II b | 2740 | 15 |  | II b | 1780 | 12 |
|  | II c | 7467 | 31 |  | II c | 9074 | 24 |
| Seawater | I | n.d. | n.d. | Seawater | I | n.d. | n.d. |
|  | II a | n.d. | n.d. |  | II a | 8664 | 80 |
|  | II b | 71348 | 379 |  | II b | 6709 | 72 |
|  | II c | 862240 | 2071 |  | II c | 10965 | 27 |

Figure S1.


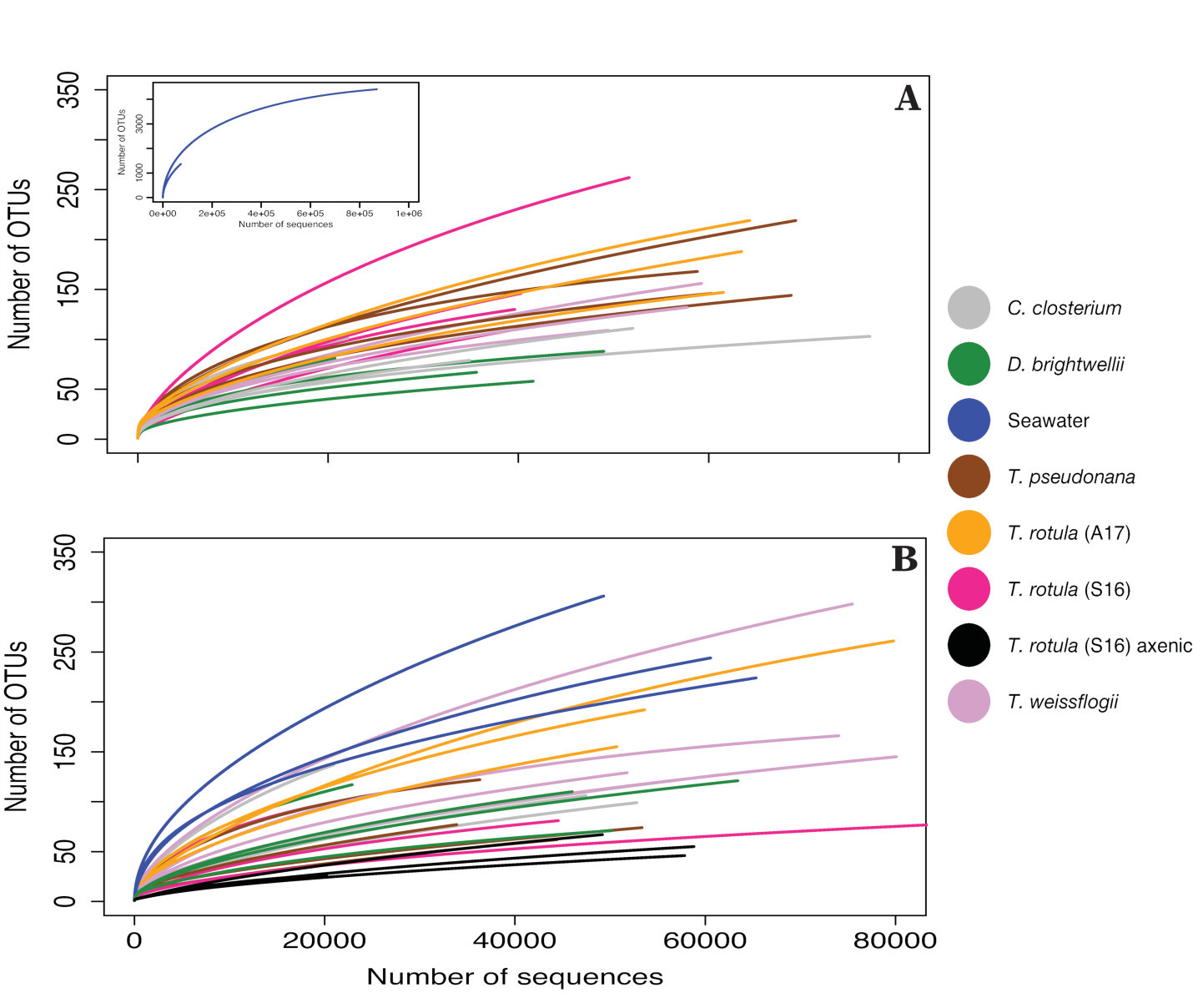


(A) Rarefaction curves of bacterial inoculum communities and (B) established *T. rotula* bacterial consortia with OTUs assigned at a 80% confidence cut-off.
